# The First National Survey of Indigenous People’s Health and Nutrition in Brazil: rationale, methodology, and overview of results

**DOI:** 10.1186/1471-2458-13-52

**Published:** 2013-01-19

**Authors:** Carlos EA Coimbra, Ricardo Ventura Santos, James R Welch, Andrey Moreira Cardoso, Mirian Carvalho de Souza, Luiza Garnelo, Elias Rassi, Maj-Lis Follér, Bernardo L Horta

**Affiliations:** 1 Escola Nacional de Saúde Pública, Fundação Oswaldo Cruz, Rua Leopoldo Bulhões 1480, Rio de Janeiro, RJ, 21041-210, Brazil; 2 Departamento de Antropologia, Museu Nacional, Universidade Federal do Rio de Janeiro, Quinta da Boa Vista s/n, Rio de Janeiro, RJ, 20940-040, Brazil; 3 Divisão de Epidemiologia, Instituto Nacional de Câncer, Rua dos Inválidos 212, Rio de Janeiro, RJ, 20231-020, Brazil; 4 Centro de Pesquisa Leônidas e Maria Deane, Fundação Oswaldo Cruz, Rua Terezina 476, Manaus, AM, 69057-070, Brazil; 5 Departamento de Saúde Coletiva, Instituto de Patologia Tropical e Saúde Pública, Universidade Federal de Goiás, Rua 235 s/n, Goiânia, GO, 74605-050, Brazil; 6 School of Global Studies, University of Gothenburg, Box 700, Göteborg, SE-405 30, Sweden; 7 Departamento de Medicina Social, Faculdade de Medicina, Universidade Federal de Pelotas, Rua Marechal Deodoro 1160, Pelotas, RS, 96001-970, Brazil

**Keywords:** Brazil, Indigenous peoples, Health surveys, Nutrition surveys, Health status indicators, Epidemiologic measurements, Maternal health, Child health

## Abstract

**Background:**

Although case studies indicate that indigenous peoples in Brazil often suffer from higher morbidity and mortality rates than the national population, they were not included systematically in any previous national health survey. Reported here for the first time, the First National Survey of Indigenous People’s Health and Nutrition in Brazil was conducted in 2008–2009 to obtain baseline information based on a nationwide representative sample. This paper presents the study’s rationale, design and methods, and selected results.

**Methods:**

The survey sought to characterize nutritional status and other health measures in indigenous children less than 5 years of age and indigenous women from 14 to 49 years of age on the basis of a survey employing a representative probabilistic sample of the indigenous population residing in villages in Brazil, according to four major regions (North, Northeast, Central-West, and South/Southeast). Interviews, clinical measurements, and secondary data collection in the field addressed the major topics: nutritional status, prevalence of hypertension and diabetes mellitus in women, child hospitalization, prevalence of tuberculosis and malaria in women, access to health services and programs, and characteristics of the domestic economy and diet.

**Results:**

The study obtained data for 113 villages (91.9% of the planned sample), 5,305 households (93.5%), 6,692 women (101.3%), and 6,128 children (93.1%). Multiple household variables followed a pattern of greater economic autonomy and lower socioeconomic status in the North as compared to other regions. For non-pregnant women, elevated prevalence rates were encountered for overweight (30.3%), obesity (15.8%), anemia (32.7%), and hypertension (13.2%). Among children, elevated prevalence rates were observed for height-for-age deficit (25.7%), anemia (51.2%), hospitalizations during the prior 12 months (19.3%), and diarrhea during the prior week (23.6%).

**Conclusions:**

The clinical-epidemiological parameters evaluated for indigenous women point to the accentuated occurrence of nutrition transition in all regions of Brazil. Many outcomes also reflected a pattern whereby indigenous women’s and children’s health indicators were worse than those documented for the national Brazilian population, with important regional variations. Observed disparities in health indicators underscore that basic healthcare and sanitation services are not yet as widely available in Brazil’s indigenous communities as they are in the rest of the country.

## Background

During the last decade international debates regarding the health of indigenous peoples have emphasized strategies for promoting health equity and reducing disparities [1-3]. Comparative analyses indicate that indigenous peoples are among the most politically and socioeconomically marginalized segments of society in the many countries in which they are present [4,5]. These disparities are reflected in inequities between indigenous and non-indigenous populations in relation to diverse health indicators, such as rates of illness and death from transmissible diseases, prevalence of child undernutrition, infant mortality rates, and years of potential life lost, all of which are generally much higher among indigenous people when compared to non-indigenous segments of the national populations where they live [6-10].

The global indigenous population is estimated at 370 million and indigenous peoples are present in about 90 countries [11]. Of this total, about 50 million live in Latin America, comprising more than 400 different ethnic groups [5]. Of all Latin American countries, Brazil has one of the smallest indigenous populations by percentage, since indigenous people make up only 0.4 percent (896,917 individuals) of the total population according to the latest national demographic census [12]. Despite the small relative size of the indigenous population in Brazil, it has enormous ethnic and linguistic diversity. Presently, as many as 300 indigenous ethnic groups, speakers of over 200 distinct languages, are present in the country [12], constituting one of the national indigenous populations with the greatest ethnic diversity in the world.

The historical advance of non-indigenous populations and economic frontiers into the interior of Brazil caused drastic depopulation and even extinction of numerous indigenous societies [13-15]. However, despite continuing high infant mortality rates and low life expectancy at birth among indigenous peoples as compared to the general population, notable demographic growth of indigenous peoples has been observed in Brazil in recent decades [16], including growth rates above the national averages [12,17].

**Figure 1:**
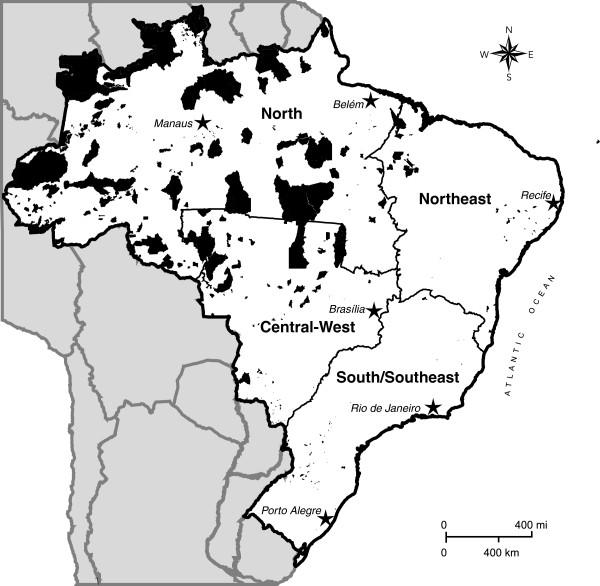
Map of Brazil indicating indigenous reserves (shaded in black) and the four geopolitical regions utilized in the First National Survey of Indigenous People’s Health and Nutrition, 2012.

At present, the majority of indigenous people in Brazil live in over 600 federally recognized reserves, 98% of which by area is located in the Amazon (Figure 1). The reserves located in the eastern and southern portions of the country tend to be smaller in size than those in the north and occur more frequently in urban settings. There is also a large indigenous contingent living outside federal reserves, both in rural and urban contexts. According to the 2010 Brazilian census, 42.3% (379,534) of the self-declared indigenous population resides outside recognized reserves [12]. Of this segment, 78.7% (298,871) lives in urban areas. In contrast, the urban segment of the indigenous population residing inside reserves is only 5.0% (25,963).

If, in recent decades, the major political challenge in Brazil was to ensure indigenous peoples full land rights, presently there also emerges the question of long-term continuity of traditional cultural systems in the face of diverse and prominent changes associated with their involvement in national society and globalization processes. With regard to indigenous subsistence and dietary practices in Brazil, diverse ethnic groups have experienced rapid transformation of economic systems in recent decades, which impacts diet and nutrition, whether through the adoption of agriculture or extractive production of goods directed to the market (coffee, rice, timber, etc.) or through paid labor [18-21].

The recent reorientation of indigenous economies has been described as causing important changes in social dynamics, food security, nutritional status, and general health [22]. In epidemiological terms, available studies point to the maintenance of high morbidity levels due to infectious and parasitic diseases (e.g., malaria, tuberculosis, diarrhea, pneumonia), much higher than those of the general Brazilian population [23-27]. Moreover, recent studies have also identified the concomitant emergence of obesity, hypertension, and diabetes mellitus in a growing number of indigenous communities in all regions of the country [19,20,28-30].

In Brazil, periodic nationwide population-based surveys conducted since the 1970s monitored not only the principal trends of morbidity and mortality, but also those related to access and use of health services by diverse socioeconomic strata of the general population. These surveys have become increasingly important for the construction of health indicators in the country. The set of information generated by these studies is recognized as fundamentally important for identifying the major health needs of different segments of the population and supporting health prevention and promotion efforts through public policy planning and evaluation [31,32]. Research based on these databases is of great interest for its potential to reveal health inequities that otherwise would remain invisible [33-35].

Considering the Brazilian government’s current agenda prioritizing the reduction of social inequities [36,37], knowledge of the health conditions, diet, and nutrition of indigenous peoples in the country is still superficial as compared to what is known about the national population, thus limiting the possibilities for characterizing and discussing their health trends. In particular, indigenous peoples in Brazil have not been adequately addressed by the major national health surveys [38]. It is noteworthy that not until 1991 did the national decennial census include the response option “indigenous” for the question about race or skin color. Also, the census only began collecting data on ethnicity and languages spoken for those who classified themselves as indigenous in 2010 [12,16].

Nevertheless, the recurrent observation of certain health conditions (e.g., high infant mortality rates, high prevalence of child undernutrition, and higher risk of tuberculosis than observed in the general population) in case studies conducted in different ethnic groups and regions indicate the vulnerability of indigenous peoples in Brazil, which places them at great disadvantage in comparison to other segments of society. Recent review articles draw attention to the difficulty of obtaining reliable and representative demographic and epidemiological data for the indigenous populations in Latin American countries, which hinders public policy formulation and prevents adequate planning and evaluation of health actions [5,39].

In order to address this lack of national health statistics for the indigenous population in Brazil, the First National Survey of Indigenous People’s Health and Nutrition (henceforth, “National Survey”) was conducted in 2008–2009. It was the first initiative to collect reliable population-based information on key health and nutrition indicators for Brazil’s indigenous peoples and is among the few of its kind in South America (for a study of comparable scope and focus in Argentina, see [40]). More commonly, demographic and health censuses in South American countries focus on specific ethnic groups rather than nationally representative samples [41-43]. The National Survey focused on the health and nutrition of indigenous women and children in all regions of Brazil in order to provide much needed information for the development of better informed national health policies.

The primary aim of this paper is to describe the rationale, design, and methods of the National Survey, as well as to present an overview of its results concerning demographic characteristics of the study population, socioeconomic and sanitation profiles of households, and the health and nutritional status of women and children. This is the first article in a series to present distinct components of the survey results. The National Survey is an important public health milestone in Brazil, as it provides for the first time information on the health and nutrition of indigenous peoples based on a nationwide representative sample. The data collected will serve as a useful resource for future evaluations of Brazil’s Indigenous Healthcare Subsystem, as well as providing a baseline for evaluating recent and future actions by the nutritional surveillance system attending the indigenous population [44]. For this reason, the findings selected for presentation in this article include health and socioeconomic indicators of comparative value relative to available data for the non-indigenous Brazilian population. Such evaluation of Brazil’s current model of indigenous healthcare is necessary for improving coverage, access, and quality of services [38,45,46].

## Methods

### Overview

As predefined by the Brazilian Ministry of Health’s National Health Foundation (Fundação Nacional de Saúde – FUNASA), the objective of the National Survey was to characterize nutritional status and other health measures in indigenous children under 5 years of age and indigenous women from 14 to 49 years of age, on the basis of a representative probabilistic sample of the indigenous population residing in Brazil.^a^ The study sample was also predesigned to be representative of the country’s official geopolitical regions. These divisions – North, Northeast, Central-West, Southeast, and South – were originally delineated on the basis of analyses of a set of environmental, economic, social, and political factors that differentiates the national territory [47]. For the purposes of the National Survey, the South and Southeast regions were joined. Thus, whereas the survey employed the four regional strata North, Northeast, Central-West, and South/Southeast, it is not possible to make inferences for specific ethnic groups, states, or administrative districts of the Indigenous Healthcare Subsystem.

Specifically, the National Survey aimed to characterize for the country as a whole and by major geopolitical region: (a) the nutritional status of children and women; (b) the prevalence of hypertension and diabetes mellitus in women; (c) the proportion of children hospitalized for diarrhea and acute respiratory infections during the prior 12 months, as reported by a parent or guardian; (d) the proportion of women reporting tuberculosis and malaria during the prior 12 months; (e) access to prenatal care, vaccination, and dietary supplementation services and programs; and (f) characteristics of the domestic economy and diet.

Realization of the National Survey was part of the implementation of the Brazilian government’s National Policy of Healthcare for Indigenous Peoples, initiated in 1999 to improve healthcare access, coverage, and quality in this population. The study was conducted by a consortium comprised of the Brazilian Public Health Association (Associação Brasileira de Saúde Coletiva – ABRASCO), which brings together public health educational and research institutions in Brazil, and the University of Gothenburg, Sweden, in collaboration with a network of researchers from universities and research institutes throughout Brazil.

### Sampling procedures

A stratified probability sample of indigenous villages in Brazil was obtained according to the regions North, Northeast, Central-West, and South/Southeast on the basis of a list provided by FUNASA on January 22, 2008. This list, the only available source of information regarding the indigenous population on a national scale, identified village populations served by the Indigenous Health Subsystem.^b^ On the date of consultation, the list contained 3,995 indigenous “villages” (“*aldeias*”) located throughout the country. Villages identified on the list as vacated (“*desaldeadas*”) or deactivated and those with less than 31 inhabitants were excluded from consideration.^c^ Although most villages were located inside federally recognized indigenous reserves, such status was not required for inclusion. Based on these criteria, 151 villages (3.8%) were excluded from the original list for being vacated or deactivated and 1,076 (26.9%) for having total populations less than 31 people. A total of 2,768 villages remained on the list for the purposes of selection.

Sample size was estimated based on the size of the target population in each region, a prevalence of 50% for all outcomes, a relative precision of 5%, and a confidence level of 95%, according to the methodology proposed by Lemeshow [48]. The presumed prevalence of 50% was chosen because this proportion maximizes the size of the sample despite a lack of overall estimates about the parameters to be investigated.

To avoid loss of precision of estimates, it was predetermined that at least 1,000 women and 1,000 children would be evaluated in each region. Furthermore, the estimated sample size was increased by 20% to avoid decreases in accuracy due to nonparticipation and other losses. It was estimated that to achieve the research objectives, it would be necessary to include at least 6,605 women and 6,583 children nationally. The numbers of villages and size of the populations of women and children eligible for the study, as well as the planned sample sizes, are presented in Table 1.

**Table 1:** Target populations of villages, women, and children (N), planned samples (n), and final samples, by region, First National Survey of Indigenous People’s Health and Nutrition, Brazil, 2008-2009

**Regions**	**Villages**				**Women**				**Children**			
					**(14–49 years)**				**(< 60 months)**			
	**N**	**Sample**			**N**	**Sample**			**N**	**Sample**		
		**n**	**Final**	**% realization**		**n**	**Final**	**% realization**		**n**	**Final**	**% realization**
**All regions**	**2768**	**123**	**113**	**91.9**	**104354**	**6605**	**6692**	**101.3**	**69259**	**6583**	**6128**	**93.1**
North	1688	65	60	92.3	44812	2720	2564	94.3	30287	2708	2584	95.4
Central-West	310	14	13	92.9	19101	1204	1295	107.6	17584	1200	1304	108.7
Northeast	586	23	22	95.7	26688	1481	1769	119.4	14232	1475	1355	91.9
South/Southeast	184	21	18	85.7	13753	1200	1064	88.7	7156	1200	885	73.8

Based on the calculated sample size for each region, villages were then selected according to the criteria of Sequential Poisson Sampling [49]. Substitution of villages was not allowed. In the end, 123 villages were selected, distributed by region as follows: 65 (North), 14 (Central-West), 23 (Northeast), and 21 (South/Southeast).

Two strategies were defined for data collection in villages: census and sample. A village was investigated by means of census when the total number of women (with or without children) between 14.0 and 49.9 years of age and children < 5 years was less than or equal to 150 individuals, according to the FUNASA list of villages. If the number of women and children in the age groups of interest was greater than 150, the village was investigated by means of a systematic sample of households. The sampling strategy combining village censuses and samples was adopted to prevent any single large village from overwhelming the results of an entire region. Although the mode of investigation (census or sample) was predetermined before the start of fieldwork, it was altered in the field for a small number of villages that were found to have actual populations that diverged greatly from those indicated on the original FUNASA list. In villages investigated by sample, households were selected according to a standardized methodology involving a predetermined increment without substitution in the event of absence or refusal. In the case of absence of residents, three visits were made before non-inclusion of a household in the final sample. All women living in selected households who identified themselves as indigenous were interviewed. Children whose mothers self-identified as indigenous and children identified as indigenous by a non-indigenous mother or caretaker were included in the study.

### Questionnaires and interviews

For the purposes of data collection, four questionnaires were applied: Village, Household, Adult Female, and Child. The first (Village) was answered by one or more community leaders. The second (Household) was answered by an adult resident, usually head-of-household. The third (Adult Female) was answered by women between 14 and 49 years of age, mothers under 14 years of age, and guardians of children under 5 years of age. Finally, the fourth (Child) was answered by a parent or caregiver of children under 5 years of age. For the sake of comparability, the questionnaires were partially based on protocols used in other population-based studies conducted in Brazil. However, given the diversity of contexts in which they would be applied in the National Survey, some items required adaptation and many additional variables were developed exclusively for the purposes of this study.

A major challenge faced during preparatory stages of the National Survey was to develop a set of questionnaires with interview questions that could be clearly communicated to and answered by respondents. Due to the enormous cultural and linguistic diversity of the population surveyed, questionnaires were written in Portuguese and local indigenous translators (most frequently indigenous health agents or primary education teachers) were used for interviewees that did not speak that language.

The Village questionnaire addressed community food production for internal consumption and for sale, including plant cultivation, animal rearing, hunting, fishing, collecting, as well as seasonality of food shortages and surpluses. The Household questionnaire included questions regarding the physical characteristics of the house, sanitation, durable household goods, sources of monetary income, food production and consumption, and seasonality of food shortages or surpluses. The Adult Female questionnaire addressed women’s education, reproductive history, current use of medications (for hypertension, diabetes, or anemia), history of illness (malaria and tuberculosis), access to prenatal care, anthropometric and clinical measurements (weight, height, arterial blood pressure, hemoglobin, and glucose levels), and vaccination history. The Child questionnaire addressed birth circumstances, access to pediatric healthcare, prior hospitalizations, recent history of diarrhea and acute respiratory infection, use of iron sulfate supplementation for anemia, breastfeeding duration, diet (for children < 2 years of age), anthropometric and clinical measurements (weight, height, and hemoglobin level), and vaccination history.

### Fieldwork and data

Fieldwork was accomplished by previously trained multidisciplinary teams. Two workshops were held to train team leaders in research procedures, methods, and instruments. Anthropometry workshops were held to standardize fieldworkers on measurement techniques. Research teams were configured according to the practical circumstances of each region and village, including population size and available forms of transportation. All teams included at least one trained leader and one person previously standardized for anthropometric measurement. In most villages, teams were accompanied by one or more local health professionals, usually nurses or nutritionists, serving each study community on behalf of FUNASA. Teams also sought the collaboration of health technicians and indigenous health agents at the FUNASA point-of-service health posts serving each village in order to facilitate interviews and the collection of secondary health data.

With very few exceptions, interviews were conducted in private households. Secondary health data were obtained from diverse records in the possession of participants or held at local or regional healthcare facilities (e.g., vaccination certificates and medical files). If possible, basic demographic information, such as sex, birthdate, and filiation, were obtained from local FUNASA healthcare records prior to conducting household interviews. Otherwise, this information was obtained from participants’ personal documents (identification cards or birth certificates) or informed by interviewees.

### Measurements

Height and weight measurements were obtained from children and women while barefoot and wearing minimum clothing. Standing height was measured with an AlturaExata portable anthropometer (Belo Horizonte, Brazil) and recorded to the nearest 0.1 cm. This anthropometer was also used to measure recumbent length of children ≤ 24 months. Body weight was measured with a portable digital floor scale with 150 kg maximum capacity and accuracy to 100 g (seca model 872, Hamburg, Germany). This scale features a mother and child weighing function that allowed children ≤ 24 months to be weighed while being held by an adult. Anthropometric measurements were obtained in accordance with Lohman [50].

Two blood pressure readings were taken from adult women 18 to 49 years of age while rested and within an interval of approximately ten to fifteen minutes using an automated wrist monitor (Omrom model HEM-631int, Bannockburn, Illinois, USA) placed on the left wrist, following standard recommendations [51]. The arithmetic mean of the two measurements was used in analyses.

To determine hemoglobin level in children and women, one drop of capillary blood obtained by fingerprick was analyzed using a portable hemoglobinometer model HemoCue Hb 201+ (Ängelholm, Sweden). Casual blood glucose level in women was assessed using an ACCU-CHECK® Active blood glucose monitoring system by Roche (Mannheim, Germany). Blood samples were collected using one-way lancets fitted to an ACCU-CHEK® lancing device.

### Data management and analysis

Data entry of questionnaire responses was done independently by two typists using Epi Info^TM^ software, version 6.04d (Centers for Disease Control and Prevention, Atlanta, GA, USA). All discrepancies between typists were researched and corrected. Data files were exported for analysis to Statistical Package for the Social Sciences (SPSS) for Windows version 16.0 (SPSS Inc., Chicago, IL, USA) and STATA 10 (College Station, TX, USA) files.

Statistics related to the characterization of the study population could be calculated without loss of precision for each of the four regions. As this was a complex sample, it was necessary to consider the effect of the study design by taking into account weights. Calculation of weights involved the relative population of each region, the relative proportions of women and children in the entire sample, and the data collection strategy adopted in each village (census or sample). To prevent statistical fluctuations, the results of calculations based on fewer than 30 responses are not reported.

The anthropometric indicators used to evaluate women’s and children’s nutrition, as well as the criteria employed to diagnose anemia, diabetes mellitus, and arterial hypertension, followed the World Health Organization (WHO) [52,53], The Seventh Report of the Joint National Committee on Detection, Evaluation, and Treatment of High Blood Pressure (JNC) [51], and The Expert Committee on the Diagnosis and Classification of Diabetes Mellitus [54], respectively.

A household goods index was generated based on the presence of durable goods in the households. A principal component analysis was carried out for 19 durable goods, considering the suitability of the data for this statistical tool. Before applying this technique, the correlation matrix between the quantities of the 19 items was calculated. Correlations varied between -0.14 (outboard motor x bicycle) and 0.58 (television x VCR/DVD). The Kaiser-Meyer-Olkin measure reached 0.81, which exceeds the minimum value (0.60) recommended for proceeding with principal component analysis. The eigenvalue of the first component of multivariate analysis was 3.56, accounting for 19% of the total variability in the dataset. The second component showed an eigenvalue 64% lower than the first, explaining 9% of the total variability.

Standing out in the first component were television set, refrigerator and/or freezer, VCR and/or DVD player, stove, telephone, and satellite dish. In the second component, outboard motor, cassava grater, and chainsaw stood out. Thus, the first component is strongly influenced by household appliances, while the second mainly includes items related to rural economic activities. Only the first component was used in defining the socioeconomic indicator, since the items in the second component disproportionally distinguished the North region. The value of the household goods index for each household is the result of the sum of the contribution of each item (generated from the principal component analysis) multiplied by the quantity of each in that household. Households were then classified according to terciles of the combined distribution, considering the four regions.

### Ethics

The National Survey was authorized by the National Research Ethics Commission (Comissão Nacional de Ética em Pesquisa – CONEP) and the National Indian Foundation (Fundação Nacional do Índio – FUNAI). All visits to villages occurred after seeking permission from community leaders via available channels of communication, such as telephone, radio, or personal communication by FUNASA health professionals. If prior permission could not be obtained due to failures of communication, permission was sought immediately upon arrival. Before initiating interviews in a given village, a meeting was held with community leaders to obtain community permission to conduct the study. To the extent possible, these meetings were held in public and formulated according to local protocols for community decision-making. In addition to describing the objectives and procedures of the study, a Free and Informed Collective Consent form was presented in detail and any questions posed by leaders or community members were answered. If consent was provided, one or more community leaders were asked to sign the form. Any particular village, household, parent, or guardian was allowed to decline to participate at any moment of fieldwork. During household visits, any additional questions about the study were answered before conducting research. Under no circumstances were individuals interviewed or measured without their consent. When possible, a community meeting was held at the conclusion of data collection in order to provide an additional opportunity for answering questions.

## Results

### Samples

Table 1 presents planned and obtained samples. Of the 123 villages in the sample, the study obtained data for 113 (91.9%). The 10 villages in the final sample that were not investigated were distributed geographically as follows: 5 in the North region, 1 in the Central-West, 1 in the Northeast, and 3 in the South/Southeast. The number of villages in which the study was conducted as compared to those planned varied from 85.7% in the South/Southeast to 95.7% in the Northeast. The non-investigation of villages was due to: refusal by village leaders to participate (3 cases); non-existence of a village at the time of research, potentially due to an error in the original FUNASA list of villages (1 case); impossibility of access due to excessive rain and flooding (1 case); access impeded by FUNASA due to H1N1 influenza epidemic (1 case); cost of access in excess of the available budget (1 case); and loss of completed research questionnaires by the postal service (1 case).

Of a total of 5,674 indigenous households originally planned for investigation, 6.5% were not interviewed, thus resulting in a total of 5,305 households investigated. The principal reason for non-inclusion was absence of the residents at the time of the field team’s visit (5.9%). It is noteworthy that only 33 households (0.6%) were not interviewed due to refusal to participate. In relation to the sex of the respondent for household questionnaires, 66% were females, with the highest percentage of female respondents being in the Northeast region (81.4%).

Overall, more than 90% of planned individual interviews (adult women and children questionnaires) were realized. The relationship between planned and realized individual interviews with women (adult female questionnaire) varied from 88.7% in the South/Southeast to 119.4% in the Northeast. As for interviews regarding children, this proportion varied from 73.8% in the South/Southeast to 108.7% in the Central-West (Table 1). The final sample exceeded that planned in the Central-West region for women (107.6%) and children (108.7%) and in the Northeast for women (119.4%). Some final sample sizes exceeded those planned because the original FUNASA population estimates were lower than those encountered during data collection. Despite losses in some regions, there was no loss of precision due to non-inclusion of adult females or children, as the allowance for non-participation in the study design was sufficient for all statistical outcomes for the entire sample and for each of the four regions.

### Sociodemographic characteristics of the sample

Table 2 presents descriptive statistics for all residents of the 113 villages investigated, as well as for women and children in the age groups of interest.

**Table 2:** Descriptive statistics for the total population and target population (women and children) of villages and households investigated, by region, First National Survey of Indigenous People’s Health and Nutrition, Brazil, 2008-2009

**Characteristics studied**	**All regions**	**Region**			
		**North**	**Central-West**	**Northeast**	**South/Southeast**
**Villages**					
**Total residents**					
Mean/SD	716.4/1304.8	360.9/659.3	2494.8/2620.5	792.9/1103.5	472.3/403.8
Min/max	30/7081	30/4642	74/7081	66/5200	81/1500
Median	268	181	1407	517	320
**Women from 14 to 49 years**					
Mean/SD	151.0/281.7	78.0/143.1	560.8/601.2	134.8/100.2	102.6/91.0
Min/max	8/1651	8/826	23/1651	14/411	14/352
Median	56	33	357	108	69
**Children < 5 years**					
Mean/SD	120.0/223.0	63.2/95.4	457.6/463.5	99.4/139.4	75.2/70.3
Min/max	5/1245	5/640	9/1245	11/606	7/252
Median	41	34	206	49	48
**Households**					
**Total residents**					
Mean/SD	6.1/3.0	7.4/3.4	5.5/2.6	5.2/2.5	5.4/2.4
Min/max	1/41	2/41	1/24	1/25	2/20
Median	6	7	5	5	5
**Women from 14 to 49 years**					
Mean/SD	1.4/0.8	1.6/1.0	1.3/0.6	1.4/0.8	1.3/0.7
Min/max	0/11	0/11	0/5	0/5	0/5
Median	1	1	1	1	1
**Children < 5 years**					
Mean/SD	1.2/1.0	1.5/1.1	1.2/1.0	1.0/1.0	1.0/1.0
Min/max	0/11	0/11	0/7	0/5	0/7
Median	1	1	1	1	1

The average total number of residents per village varied from 360.9 (North region) to 2,494.8 (Central-West), while the medians varied from 181 (North) to 1,407 (Central-West). In general, sampled villages were small, with over half having less than 268 inhabitants. Few villages had populations surpassing 7,000, as observed in the Central-West, which was the region with the largest villages in the sample. Consistent with these findings, the distribution of women and children by region (Table 2) showed the largest contingents by village in the Central-West region.

Table 2 shows that the mean number of household residents varied from 5.2 in the Northeast to 7.4 in the North. The maximum number of residents varied greatly, from 20 in the South/Southeast to 41 in the North. Important differences were also observed by region in the number of women and children in sampled households, with the North presenting the highest values for both (11 women and 11 children).

The age distribution of sampled children was relatively balanced between regions (Table 3). Approximately 20% of sampled children were distributed in each 12 month interval. For the entire sample, as for each of the regions, the number of boys was slightly higher than girls, especially in the Northeast and Central-West regions.

**Table 3:** Distribution of indigenous children < 60 months by age group and sex, by region, First National Survey of Indigenous People’s Health and Nutrition, Brazil, 2008-2009

**Characteristics**	**All regions**	**Region**			
**studied**		**North**	**Central-West**	**Northeast**	**South/Southeast**
**Age group**	n = 6128	n = 2584	n = 1304	n = 1355	n = 885
0 to 11 months	21.7%	22.4%	22.1%	20.5%	21.6%
	CI:20.5-22.9	CI:21.2-23.7	CI:20.2-24.1	CI:18.7-22.5	CI:18.2-25.5
12 to 23 months	19.3%	21.5%	17.0%	19.4%	19.3%
	CI:18.0-20.6	CI:19.9-23.1	CI:13.9-20.6	CI:16.8-22.3	CI:16.6-22.4
24 to 35 months	19.2%	19.3%	20.3%	19.4%	18.3%
	CI:18.2-20.3	CI:17.5-21.1	CI:18.4-22.3	CI:17.0-22.0	CI:16.2-20.7
36 to 47 months	20.8%	19.4%	19.6%	23.0%	20.8%
	CI:19.7-21.8	CI:18.1-20.8	CI:18.4-20.8	CI:21.6-24.5	CI:18.1-23.9
48 to 59 months	19.1%	17.4%	21.1%	17.6%	19.9%
	CI:17.7-20.5	CI:16.3-18.6	CI:18.2-24.3	CI:16.1-19.3	CI:16.3-24.1
**Sex**	n = 6127	n = 2584	n = 1303	n = 1355	n = 885
Male	51.4%	50.7%	52.5%	52.3%	50.2%
	CI:49.8-52.9	CI:49.1-52.3	CI:50.3-54.7	CI:49.3-55.3	CI:45.9-54.5
Female	48.6%	49.3%	47.5%	47.7%	49.8%
	CI:47.1-50.2	CI:47.7-50.9	CI:45.3-49.7	CI:44.7-50.7	CI:45.5-54.1

*CI*: 95% confidence interval.

Approximately a third of women interviewed (38.4%) were between 20 and 29 years of age (Table 4), varying from 36.7% in the North to 39.9% in the Northeast. In total, 15.2% of women had no formal education, reaching 21.9% in the Central-West region. The greater proportion of women reported having between 1 and 4 years of schooling (38.0%), varying from 34.4% in the South/Southeast to 43.8% in the North. The highest percentages of women with 10 or more years of schooling were observed in the Northeast (29.7%) and South/Southeast (19.3%).

**Table 4:** Distribution of indigenous women from 14 to 49 years by age group and schooling, by region, First National Survey of Indigenous People’s Health and Nutrition, Brazil, 2008-2009

**Characteristics**	**All regions**	**Region**			
**studied**		**North**	**Central-West**	**Northeast**	**South/Southeast**
**Age group**	n = 6692	n = 2564	n = 1295	n = 1769	n = 1064
14 to 19 years	25.2%	26.1%	25.2%	22.9%	26.3%
	CI:23.9-26.5	CI:24.0-28.3	CI:22.9-27.7	CI:20.2-25.9	CI:23.6-29.2
20 to 29 years	38.4%	36.7%	39.9%	39.5%	37.7%
	CI:36.8-40.1	CI:34.4-39.0	CI:36.0-43.9	CI:36.0-43.0	CI:34.1-41.4
30 to 39 years	24.1%	25.5%	23.7%	25.2%	22.6%
	CI:23.0-25.3	CI:23.6-27.5	CI:20.8-26.9	CI:23.2-27.3	CI:20.5-25.0
40 to 49 years	12.3%	11.7%	11.2%	12.4%	13.4%
	CI:11.3-13.3	CI:10.3-13.2	CI:9.9-12.7	CI:9.9-15.5	CI:11.5-15.4
**Schooling**	n = 6643	n = 2523	n = 1294	n = 1766	n = 1060
None	15.2%	16.5%	21.9%	8.2%	14.6%
	CI:12.1-18.8	CI:11.5-23.2	CI:14.8-31.1	CI:4.8-13.8	CI:.9.0-22.9
1-4 years	38.0%	43.8%	39.0%	36.4%	34.4%
	CI:35.1-40.9	CI:39.0-48.7	CI:33.4-45.0	CI:28.9-44.7	CI:29.6-39.5
5-9 years	28.3%	27.1%	27.8%	25.6%	31.7%
	CI:25.8-30.9	CI:23.0-31.7	CI:21.7-34.8	CI:21.0-31.0	CI:26.6-37.2
10 years or +	18.6	12.6	11.3	29.7	19.3
	CI:15.0-22.9	CI:9.0-17.3	CI:6.4-19.0	CI:21.8-39.1	CI:12.9-27.9

*CI*: 95% confidence interval.

### Household characteristics

Substantial differences between regions were observed in household construction materials used for flooring, walls, and roofing (Table 5). In the North, wood was the predominant material for flooring and walls (55.6% and 64.0%, respectively) and corrugated zinc/asbestos sheets and wood or thatch (52.6% and 46.3%, respectively) were the most common for roofing. In contrast, the predominant construction materials used in the Northeast were industrialized: ceramic or cement for flooring (82.4%), bricks for walls (76.5%), and roofing of clay tiles (81.3%).

**Table 5:** Distribution of households by infrastructure characteristics (flooring, walls and roofing) and household goods index, by region, First National Survey of Indigenous People’s Health and Nutrition, Brazil, 2008-2009

**Characteristics studied**	**All regions**	**Region**			
		**North**	**Central-West**	**Northeast**	**South/Southeast**
**Type of flooring**	n = 5251	n = 1826	n = 1083	n = 1451	n = 891
Ceramic or cement	46.0%	15.8%	41.7%	82.4%	53.8%
	CI:44.6-47.3	CI:14.2-17.5	CI:38.8-44.7	CI:80.4-84.3	CI:50.5-57.0
Wood	23.1%	55.6%	0.1%	0.1%	22.1%
	CI:22.0-24.3	CI:53.3-57.9	CI:0.0-0.3	CI:0.0-0.2	CI:19.4-24.8
Dirt	30.9%	28.6%	58.2%	17.6%	24.1%
	CI:29.6-32.1	CI:26.5-30.7	CI:55.2-61.1	CI:15.6-19.5	CI:21.3-26.9
**Type of walls**	n = 5270	n = 1832	n = 1083	n = 1462	n = 893
Brick	41.8%	10.5%	45.3%	76.5%	44.8%
	CI:40.4-43.1	CI:9.1-11.9	CI:42.4-48.3	CI:74.3-78.6	CI:41.5-48.1
Wood	32.4%	64.0%	21.5%	1.3%	31.9%
	CI:31.2-33.7	CI:61.8-66.2	CI:19.1-24.0	CI:0.7-1.9	CI:28.9-35.0
Thatch or mud	16.8%	22.7%	8.3%	20.0%	9.9%
	CI:15.8-17.8	CI:20.8-24.6	CI:6.7-10.0	CI:18.0-22.1	CI:7.9-11.8
Canvas, plastic, or other	9.0%	2.8%	24.8%	2.2%	13.4%
	CI:8.2-9.7	CI:2.0-3.5	CI:22.3-27.4	CI:1.4-2.9	CI:11.2-15.7
**Type of roofing**	n = 5273	n = 1832	n = 1083	n = 1463	n = 895
Clay tiles	36.9%	1.0%	22.4%	81.3%	55.5%
	CI:35.6-38.2	CI:0.5-1.4	CI:19.9-24.9	CI:79.3-83.3	CI:52.3-58.8
Corrugated zinc/asbestos sheets	36.9%	52.6%	46.2%	8.6%	39.8%
	CI:35.6-38.2	CI:50.3-55.0	CI:43.2-49.1	CI:7.2-10.1	CI:36.6-43.0
Wood or thatch	25.4%	46.3%	30.2%	9.8%	2.6%
	CI:24.3-26.6	CI:44.0-48.6	CI:27.5-32.9	CI:8.3-11.3	CI:1.5-3.6
Canvas, plastic, or other	0.7%	0.2%	1.2%	0.3%	2.1%
	CI:0.5-1.0	CI:0.0-0.3	CI:0.6-1.8	CI:0.0-0.5	CI:1.2-3.1
**Household goods index**	n = 5283	n = 1839	n = 1083	n = 1464	n = 897
1º Tercile	33.4%	50.8%	38.4%	14.6%	22.2%
	CI:32.1-34.7	CI:48.6-53.1	CI:35.5-41.3	CI:12.8-16.4	CI:19.5-24.9
2º Tercile	33.2%	33.6%	33.8%	34.9%	29.1%
	CI:31.9-34.5	CI:31.4-35.7	CI:31.0-36.6	CI:32.5-37.3	CI:26.1-32.1
3º Tercile	33.4%	15.6%	27.8%	50.5%	48.7%
	CI:32.1-34.7	CI:13.9-17.3	CI:25.1-30.5	CI:47.9-53.0	CI:45.4-52.0

*CI*: 95% confidence interval.

The distribution of the household goods index (Table 5) used as a proxy for socioeconomic status shows that the North was the region with the greatest proportion of households in the lower tercile (50.8%). In contrast, in the Northeast and South/Southeast regions, the greatest proportions of households were in the upper tercile of the distribution (50.5% and 48.7%, respectively).

With regard to sanitation, the residents of 19.4% of households reported defecating in facilities inside their homes (e.g., bathrooms), 49.5% in detached outdoor facilities (e.g., latrines), and 30.6% in the open (Table 6). By region, the Northeast and South/Southeast presented the highest frequencies of households with some kind of sanitary installation inside the house (45.4% and 25.7%, respectively). In contrast, in the North just 1.0% of households had indoor installations. For all regions, the predominant form of waste disposal in households with some kind of sanitary facility either inside or outside the house was rudimentary pit latrine (63.3%), ranging from 28.8% in the South/Southeast to 91.0% in the North (Table 6).

**Table 6:** Distribution of households by sanitary conditions and presence of electricity, by region, First National Survey of Indigenous People’s Health and Nutrition, Brazil, 2008-2009

**Characteristics studied**	**All regions**	**Region**			
		**North**	**Central-West**	**Northeast**	**South/Southeast**
**Defecation location**	n = 5268	n = 1827	n = 1083	n = 1463	n = 895
Indoor household facility	19.4%	1.0%	10.0%	45.4%	25.7%
	CI:18.3-20.5	CI:0.6-1.5	CI:8.2-11.8	CI:42.9-48.0	CI:22.8-28.6
Outdoor household facility	49.5%	58.2%	71.2%	29.5%	38.1%
	CI:48.1-50.8	CI:55.9-60.4	CI:68.5-73.9	CI:27.1-31.8	CI:34.9-41.3
Outdoors in the open	30.6%	40.4%	18.6%	23.9%	35.9%
	CI:29.3-31.8	CI:38.2-42.7	CI:16.2-20.9	CI:21.7-26.0	CI:32.7-39.0
Other	0.6%	0.3%	0.3%	1.2%	0.3%
	CI:0.4-0.8	CI:0.1-0.6	CI:0.0-0.6	CI:0.7-1.8	CI:0.0-0.7
**Predominant destination of human waste**	n = 3567	n = 1041	n = 875	n = 1085	n = 566
Sewage disposal system	5.9%	0%	0.2%	14.8%	8.3%
	CI:5.1-6.6	–	CI:0.0-0.5	CI:12.6-16.9	CI:6.0-10.6
Septic system	30.6%	8.7%	26.3%	38.3%	62.5%
	CI:29.1-32.1	CI:7.0-10.5	CI:23.4-29.2	CI:35.4-41.2	CI:58.5-66.5
Rudimentary pit latrine	63.3%	91.0%	73.5%	46.6%	28.8%
	CI:61.7-64.9	CI:89.2-92.7	CI:70.6-76.4	CI:43.7-49.6	CI:25.1-32.5
River, lake, or ocean	0.2%	0.3%	0%	0.3%	0.4%
	CI:0.1-0.4	CI:0.0-0.6	–	CI:0.0-0.6	CI:0.0-0.8
**Predominant destination of household trash**	n = 5271	n = 1831	n = 1083	n = 1463	n = 894
Collected by removal service	15.0%	1.1%	0%	38.4%	23.3%
	CI:14.0-16.0	CI:0.6-1.6	–	CI:35.9-40.9	CI:20.5-26.0
Buried, discarded, or burned in the village	79.0%	85.9%	98.6%	58.6%	74.7%
	CI:77.9-80.1	CI:84.3-87.5	CI:97.9-99.3	CI:56.0-61.1	CI:71.9-77.6
Discarded in a river, lake, or ocean	0.8%	2.2%	0%	0.2%	0%
	CI:0.6-1.1	CI:1.5-2.9	–	CI:0.0-0.4	–
Other	5.2%	10.8%	1.4%	2.8%	2.0%
	CI:4.6-5.8	CI:9.4-12.2	CI:0.7-2.1	CI:2.0-3.6	CI:1.1-2.9
**Predominant source of drinking water**	n = 5236	n = 1799	n = 1082	n = 1461	n = 894
Municipal system	9.4%	0.2%	0.1%	28.0%	8.6%
	CI:8.6-10.1	CI:0.0-0.4	CI:0.0-0.3	CI:25.7-30.3	CI:6.8-10.5
Spring or artesian well	55.2%	36.3%	87.5%	40.4%	78.4%
	CI:53.9-56.6	CI:34.1-38.5	CI:85.6-89.5	CI:37.9-42.9	CI:75.7-81.1
Shallow well	8.6	13.3	3.8	6.2	8.6
	CI:7.8-9.3	CI:11.8-14.9	CI:2.6-4.9	CI:4.9-7.4	CI:6.8-10.5
River, lake or reservoir	11.6%	28.1%	4.8%	1.8%	2.3%
	CI:10.7-12.4	CI:26.0-30.1	CI:3.5-6.1	CI:1.2-2.5	CI:1.4-3.3
Other	15.3%	22.1%	3.8%	23.6%	2.0%
	CI:14.3-16.3	CI:20.2-24.0	CI:2.6-4.9	CI:21.4-25.8	CI:1.1-2.9
**Electricity in the home**	n = 5266	n = 1827	n = 1083	n = 1461	n = 895
Yes	64.8%	30.1%	66.4%	94.4%	85.6%
	CI:63.5-66.1	CI:28.0-32.2	CI:63.6-69.2	CI:93.2-95.6	CI:83.3-87.9
Yes, but discontinuous	12.8%	34.5%	2.7%	0.4%	0.9%
	CI:11.9-13.7	CI:32.4-36.7	CI:1.7-3.6	CI:0.1-0.7	CI:0.3-1.5
No	22.4%	35.4%	30.9%	5.2%	13.5%
	CI:21.2-23.5	CI:33.2-37.6	CI:28.2-36.7	CI:4.1-6.3	CI:11.3-15.8

*CI*: 95% confidence interval.

Considering all regions, 79.0% of households reported disposing of domestic trash in the village, either being buried, burned, or discarded, often in the peridomicile (Table 6). In general, access by indigenous households to trash collection services was restricted, being observed more frequently in the Northeast (38.4%) and South/Southeast (23.3%).

Considering all regions together, the most commonly reported primary sources of drinking water were those located in the immediate vicinities of villages, such as natural springs and artesian wells (Table 6). This category presented strong variation between regions, ranging from 36.3% in the North to 87.5% in the Central-West. The North region was distinguished from the others by presenting greater frequencies of shallow wells (13.3%) and rivers, lakes, or reservoirs (28.1%) as important sources of drinking water. In contrast, the Northeast showed the highest rates of water deriving from public municipal networks (28.0%) and other sources, such as trucks or ponds (23.6%).^d^

Nationwide, 77.6% of households reported having electricity, with 64.8% having continuous sources (public or private utilities) and 12.8% discontinuous (private or community generators). Notable contrasts in the presence of domestic electricity were observed between regions. Whereas the proportion of households without electricity was only 5.2% in the Northeast, it reached 35.4% in the North and 30.9% in the Central-West.

Although differences were observed between regions in how foods were obtained, almost all households in the nationwide sample (96.4%) reported routinely purchasing at least one food item (Table 7). In the North, a slightly lower proportion of households reported purchasing at least some food (91.8%) than in the other regions. The second most frequently reported means of acquiring foods was plant cultivation and animal rearing, which presented relative homogeneity between all regions except the Northeast, where the prevalence was significantly lower (71.7%). The proportions of households reporting acquiring foods by collecting ranged from 58.2% (South/Southeast) to 85.4% (North), and by hunting or fishing from 45.0% (Central-West) to 94.0% (North). Obtaining food by means of government distribution of “basic food baskets” (*cestas básicas*) presented some disparity between regions. Whereas the greater proportions of indigenous households in the Central-West (88.6%) and South/Southeast (77.8%) received food by this means, this figure was significantly lower in the Northeast (30.6%) and North (3.5%). Although other food sources (e.g., donations by churches, NGOs, and others) were reported much less frequently in all regions, these were somewhat less prevalent in the North (8.1%).

**Table 7:** Distribution of principle food sources reported by indigenous households, by region, First National Survey of Indigenous People’s Health and Nutrition, Brazil, 2008-2009

**Characteristics studied**	**All regions**	**Region**			
		**North**	**Central-West**	**Northeast**	**South/Southeast**
	n = 5273	n = 1832	n = 1083	n = 1463	n = 895
Cultivation and/or raising animals	83.2%	89.4%	87.4%	71.7%	84.6%
	CI:82.2-84.2	CI:87.9-90.8	CI:85.4-89.3	CI:69.4-74.0	CI:82.2-87.0
Hunting or fishing	64.8%	94.0%	45.0%	52.9%	48.3%
	CI:63.5-66.0	CI:92.9-95.1	CI:42.0-47.9	CI:50.3-55.5	CI:45.0-51.5
Collecting	69.0%	85.4%	62.2%	60.0%	58.2%
	CI:67.7-70.2	CI:83.8-87.0	CI:59.3-65.1	CI:57.5-62.5	CI:55.0-61.4
Purchase	96.4%	91.8%	97.9%	99.2%	99.1%
	CI:95.8-96.9	CI:90.6-93.1	CI:97.0-98.7	CI:98.8-99.7	CI:98.5-99.7
Basic food baskets	41.1%	3.5%	88.6%	30.6%	77.8%
	CI:39.8-42.4	CI:2.6-4.3	CI:86.7-90.5	CI:28.3-33.0	CI:75.0-80.5
Other donations (churches, NGOs and others)	13.5%	8.1%	16.3%	17.0%	15.8%
	CI:12.6-14.5	CI:6.8-9.3	CI:14.1-18.5	CI:15.0-18.9	CI:13.4-18.1

*CI*: 95% confidence interval.

### Women’s health

Nationwide, 46.1% of non-pregnant indigenous women had excess weight (30.3% overweight and 15.8% obese) following WHO cut-off points for the interpretation of Body mass index (BMI) [52]. Notably, comparing the North with the other regions, the frequencies of women classified as overweight or obese were markedly different. In the South/Southeast and Central-West, obesity prevalence rates reached 22.6% and 17.2%, respectively, exceeding that observed in the North by 3.7 and 2.8 times, respectively.

The prevalence of anemia in non-pregnant women according to WHO diagnosis criteria [53] was elevated (32.7%), with important differences between regions (Table 8). The North was the region with the highest frequency of anemia (46.8%). In this region, the prevalence of anemia exceeded by 1.4 times the value observed among indigenous women nationally.

**Table 8:** Prevalence rates of underweight, overweight, obesity, anemia, hypertension, and diabetes mellitus in indigenous women from 14 to 49 years and principle results related to prenatal exam of the youngest child < 60 months, by region, First National Survey of Indigenous People’s Health and Nutrition, Brazil, 2008-2009

**Characteristics Studied**	**All regions**	**Region**			
		**North**	**Central-West**	**Northeast**	**South/Southeast**
Underweight ^1^	n = 5714	n = 2064	n = 1112	n = 1585	n = 953
	2.4%	2.4%	0.9%	3.6%	2.5%
	CI:1.7-3.2	CI:1.6-3.4	CI:0.4-2.2	CI:2.5-5.2	CI:1.3-4.6
Overweight ^1^	n = 5714	n = 2064	n = 1112	n = 1585	n = 953
	30.3%	24.7%	35.3%	27.7%	32.0%
	CI:28.2-32.4	CI:20.8-29.5	CI:32.7-38.0	CI:25.1-30.5	CI:27.6-36.7
Obesity ^1^	n = 5714	n = 2064	n = 1112	n = 1585	n = 953
	15.8%	6.1%	17.2%	13.5%	22.6%
	CI:12.5-19.8	CI:3.4-10.7	CI:13.0-22.3	CI:10.4-17.5	CI:14.5-33.4
Anemia ^2^	n = 5720	n = 2068	n = 1116	n = 1586	n = 950
	32.7%	46.8%	34.9%	22.6%	30.6%
	CI:29.7-35.8	CI:41.4-52.3	CI:31.4-38.5	CI:18.1-27.8	CI:25.2-36.5
Hypertension ^3^	n = 4753	n = 1679	n = 942	n = 1347	n = 785
	13.2%	3.6%	17.5%	11.2%	17.4%
	CI:11.2-15.5	CI:2.3-5.6	CI:13.9-21.9	CI:7.8-15.9	CI:15.0-20.1
Diabetes mellitus ^4^	n = 5722	n = 2070	n = 1115	n = 1587	n = 950
	1.4%	0.5%	1.4%	1.1%	2.1%
	CI:1.0-1.9	CI:0.3-1.0	CI:0.9-2.1	CI:0.6-2.2	CI:1.2-3.6
At least 1 prenatal consultation during first trimester ^5^	n = 2427	n = 792	n = 642	n = 578	n = 415
	46.1%	33.4%	43.3%	58.6%	47.2%
	CI:41.3-51.0	CI:27.3-40.1	CI:34.1-53.1	CI:53.0-63.9	CI:37.8-56.7
At least six prenatal consultations ^5^	n = 2549	n = 848	n = 654	n = 586	n = 461
	36.4%	10.9%	33.3%	48.3%	45.0%
	CI:31.0-42.1	CI:7.4-16.0	CI:24.5-43.5	42.1-54.5	CI:35.2-55.2

*CI*: 95% confidence interval.

^1^ Underweight (BMI < 18.5 kg/m^2^), overweight (BMI 25–29.9), and obesity (BMI ≥ 30) in non-pregnant women [52].

^2^ Hemoglobin concentration < 12.0 mg/dL in non-pregnant women [53].

^3^ Sistolic blood pressure (BP) ≥ 140 and/or diastolic BP ≥ 90 mmHg, according to the JNC 7 report [51] in non-pregnant women. Prevalence rates include women who reported current use of anti-hypertension medication as members of the group with elevated BP, independent of observed BP levels.

^4^ Blood glucose ≥ 200 mg/dl in non-pregnant women [54].

^5^ Proportions calculated with reference to women with at least one prenatal consultation with a nurse or doctor during the pregnancy with the youngest living child < 60 months of age.

The prevalence of hypertension following the JNC seventh report [51], was 13.2% for the entire sample of non-pregnant women (Table 8). The frequency of women with high blood pressure in the four regions was heterogeneous, with the prevalence in the North (3.6%) being significantly lower than in the other regions. In contrast, the prevalence of hypertension was approximately three times higher in the Northeast and five times higher in the South/Southeast and Central-West than in the North.

The prevalence of blood glucose values suggestive of diabetes mellitus in non-pregnant women was 1.4%, varying from 0.5% in the North to 2.1% in the South/Southeast (Table 8).

Prenatal consultation with a nurse or doctor during the first trimester of pregnancy with one’s youngest living child less than five years of age was reported by 46.1% of women nationwide, ranging from 33.4% in the North to 58.6% in the Northeast (Table 8). Only 36.4% of women nationwide had at least six prenatal consultations during this pregnancy, ranging from 10.9% in the North to 48.3% in the Northeast.

### Children’s health

The survey results regarding children less than five years of age show elevated prevalence rates of low height-for-age according to the growth curve reference published by the WHO [52] (25.7%), varying from 13.9% in the Northeast to 40.8% in the North. Additionally, 51.2% of indigenous children nationally were found to be anemic, varying from 41.1% in the Northeast to 66.4% in the North (Table 9).

**Table 9:** Prevalence rates of low height-for-age and anemia in indigenous children < 60 months, proportion of children with reported hospitalization during the previous year, proportions of reported hospitalization due to diarrhea and acute respiratory infection and proportion with reported diarrhea during the previous week, by region, First National Survey of Indigenous People’s Health and Nutrition, Brazil, 2008-2009

	**Characteristics studied**	**All regions**	**Region**			
			**North**	**Central-West**	**Northeast**	**South/Southeast**
Low height-for-age ^1^	n = 6011	n = 2539	n = 1277	n = 1331	n = 864
	25.7%	40.8%	27.6%	13.9%	22.3%
	CI:21.8-30.0	CI:35.7-46.2	CI:20.4-36.1	CI:10.3-18.6	CI:14.2-33.3
Anemia ^2^	n = 5397	n = 2280	n = 1141	n = 1211	n = 765
	51.2%	66.4%	51.5%	41.1%	48.0%
	CI:47.9-54.6	CI:61.6-70.9	CI:45.7-57.3	CI:34.4-48.0	CI:40.6-55.4
Hospitalized during prior year	n = 6087	n = 2555	n = 1298	n = 1352	n = 882
	19.3%	16.9%	27.3%	14.0%	19.2%
	CI:16.6-22.3	CI:12.2-22.9	CI:22.2-33.1	CI:11.0-17.6	CI:15.2-24.0
With at least one hospitalization due to diarrhea during prior year	n = 1117	n = 426	n = 347	n = 181	n = 163
	37.2%	48.4%	43.5%	24.8%	29.8%
	CI:32.5-42.1	CI:39.9-56.9	CI:36.6-50.7	CI:16.7-35.3	CI:21.9-39.1
With at least one hospitalization due to acute respiratory infection during prior year	n = 1115	n = 425	n = 348	n = 180	n = 162
	47.6%	54.4%	40.4%	35.6%	58.4%
	CI:41.8-53.5	CI:45.2-63.2	CI:33.0-48.1	CI:27.6-44.4	CI:43.6-71.8
With diarrhea during the prior week	n = 6068	n = 2546	n = 1298	n = 1347	n = 877
	23.6%	38.1%	21.4%	19.4%	17.8%
	CI:20.9-26.4	CI:34.6-41.8	CI:18.6-24.4	CI:14.9-24.9	CI:12.8-24.2

*CI*: 95% confidence interval.

^1^ Low height-for-age according to WHO standards [52].

^2^ Hemoglobin concentration < 11.0 mg/dL in children > 6 months of age [53].

The nationwide proportion of children with reported hospitalizations during the prior 12 months (19.3%) was elevated (Table 9). This prevalence was highest in the Central-West (27.3%). Diarrhea and acute respiratory infection were frequent causes of hospitalization. With respect to referred morbidity during the prior week, about one in four children (23.6%) presented diarrhea. In the North, the proportion of children presenting diarrhea during the prior week (38.1%) was significantly higher as compared to the other regions.

## Discussion

This article presents the design and methodology used in the First National Survey of Indigenous People’s Health and Nutrition conducted in Brazil and reports on general characteristics of the population and households investigated, as well as the nutrition and health profiles of indigenous women and children. The study included a total sample of 113 villages, 5,305 households, 6,692 women, and 6,128 children. The scope of the survey was similar to population-based studies previously conducted for Brazil’s non-indigenous population, such as a recent 2006 national survey of women 15–49 years of age and children less than 5 years of age, which had a sample size of approximately 15,000 women and 5,000 children [55]. In addition to examining and interviewing a large number of indigenous individuals, the National Survey involved complex logistical planning in order to reach more distant and isolated communities, especially in the North and Central-West regions, many of which could only be accessed by riverboats, small airplanes, or four-wheel drive vehicles.

Considered generally, the results of the National Survey reveal marked differences between the indigenous populations in the country’s major geopolitical regions for many of the variables investigated. In part, these contrasts are associated with the regional histories of Brazil’s expanding demographic and economic frontiers and how these affected indigenous peoples. An interesting aspect of this historical process involves the regional distribution of indigenous reserves, as mentioned in the introduction to this article (Figure 1). About 13% of the Brazilian territory is recognized as federal indigenous land [56]. Of this, 98% by area is located in the Amazon (North region and the northern portion of the Central-West region) and the remaining 2% is in the other portions of the country. The indigenous reserves in the northern portion of the country tend to be much larger than those in the coastal Northeast and South/Southeast regions, which were the first to be colonized by Europeans [13,57]. The indigenous peoples living in these regions often suffered larger territorial losses as a result of early depopulation and historical policies unfavorable to the recognition of their land rights. The colonization of the country subsequently progressed westward and northward, with encroachment of indigenous lands in the Brazilian Amazon region generally occurring more recently, often as late as recent decades when the country’s more favorable policies for recognizing indigenous lands resulted in larger indigenous reserves.

The findings of the National Survey regarding regional patterns of dietary subsistence in indigenous households are likely related to the historical contrasts in the country’s distribution of indigenous lands. As described in the Methods, most of the villages sampled were located in indigenous reserves, although this was not a selection criterion. Accordingly, in the North, the region with the greatest extension of indigenous lands, a larger proportion of indigenous households reported consuming foods obtained from cultivation and animal raising, hunting and fishing, and collecting. Foods obtained by these productive activities were reported less frequently for households in the Northeast and South/Southeast regions. These inter-regional differences in subsistence patterns may partially derive from the regional heterogeneity of access to land with the potential to support indigenous food production activities.

The distribution of materials used for roofing, walls, and flooring in indigenous houses follows a similar pattern. Whereas locally produced materials, such as wood and thatch, predominated in the North, industrialized materials, such as cement, clay tiles, and corrugated zinc/asbestos sheets, were found to be more common in the Northeast and South/Southeast.

Although the observed regional distributions of food production and house construction may partially reflect contrasting patterns of territorial access to natural resources in indigenous reserves, they also appear related to disparate socioeconomic conditions. Households in the North region not only utilize proportionally less industrialized materials in their physical construction, but also have less access to electricity and present lower socioeconomic scores, as measured by the household goods index, which was strongly influenced by electric appliances. Reiterating the pattern of interregional heterogeneity mentioned above, households in the Northeast and South/Southeast regions had the highest socioeconomic scores. Similarly, the results indicate that indigenous women in the North and Central-West regions tend to have less years of schooling than those residing in the Northeast and South/Southeast.

Effective human waste disposal is considered a key intervention in disease prevention and environmental health [58-60]. The results of the National Survey highlight major gaps in the availability of public services to indigenous villages such as basic sanitation, safe drinking water, and waste management. The most typical human waste disposal infrastructure observed in the sample was that of a simple pit latrine, with sewage rarely being collected or receiving any treatment. Even in those regions with higher socioeconomic scores, substantial proportions of interviewees reported that their household members defecate in the open, as in the South/Southeast (35.9%). Household trash management was also found to be precarious, with trash most commonly being buried, discarded, or burned in the peridomicile or elsewhere in the village. Only in the Northeast was there greater access to public garbage collection services, which reached 38.4% of households in this region.

These findings sharply contrast with the non-indigenous population in Brazil. According to the 2008 National Basic Sanitation Survey [61], 91.8% of households located in Brazil’s rural areas had human waste disposal infrastructure, ranging from 84.8% in the Northeast to 99.1% in the South. With respect to garbage collection, 42.4% of Brazilian rural households had access to public services, varying from 24.2% in the North to 63.0% in the South [61].

The profile of sanitation conditions outlined by the National Survey reveals marked inequities between indigenous and non-indigenous households in Brazil, with the indigenous population being strongly disadvantaged with regard to access to water, sanitation infrastructure, and management of solid waste. It is important to note that the results of the National Survey show that many villages, particularly in the Central-West region, have artesian well water supply systems, often installed by FUNASA. These are part of an important governmental initiative aimed at expanding the sanitation network in indigenous communities. However, despite the widespread presence of such wells, interviews with indigenous leaders during the survey fieldwork commonly revealed complaints that problems persisted with their daily functioning, such as insufficient water tank capacities, broken valves or pumps, and nonexistent connections between dug wells and installed water tanks, among others.

The epidemiological parameters evaluated for indigenous women point to the accentuated occurrence of nutrition transition in all regions of Brazil. In total, 30.3% of indigenous women were classified as overweight and 15.8% as obese. There were also important inter-regional differences, with women in the North region presenting lower prevalence rates of overweight and obesity than those in the other regions. Previous case studies from different indigenous communities in Brazil have documented similarly high prevalence rates of overweight and obesity in adults. For example, obesity was found to affect 22.2% of women among the Suruí, 24.5% among the Xavante, and 30.8% among the Guarani-Kaiowá [19,20,62]. Whereas the prevalence of obesity observed among indigenous women in the National Survey is comparable to that of non-indigenous Brazilian women 15–49 years (16.1%), non-indigenous women have a higher prevalence of overweight (43.1%) according to the 2006 National Demography and Health Survey [55].

The overall prevalence rates of hypertension among indigenous women was 13.2%, with higher values in the Central-West (17.5%) and South/Southeast (17.4%). According to the results of the most recent health survey based on a representative sample of the national non-indigenous population, 24.0% of Brazilian women presented hypertension [63]. The prevalence of anemia among non-pregnant indigenous women was also elevated, affecting approximately one in three women in the overall sample. In comparison, the prevalence of anemia among non-indigenous women in Brazil is slightly lower (29.4%) [55].

Case studies conducted in specific indigenous communities in Brazil since the 1990s, mainly in the North and Central-West regions, call attention to high prevalence rates of chronic undernutrition among indigenous children, characterized by linear growth deficits [21,64-68]. The results of the National Survey confirm on a national scale that chronic undernutrition, as measured by low height-for-age, is in fact a problem of great magnitude, affecting one in four indigenous children in Brazil. In addition, approximately half of indigenous children in Brazil were found to have anemia. In contrast, the information available for non-indigenous Brazilian children in the same age group indicates lower prevalence rates of anemia (20.9%) and of low height-for-age (7.1%) [33,55]. The nutritional profile of the overall child population in Brazil has been interpreted favorably as resulting from the recent implementation of a universal public healthcare system, as well as increased sanitation coverage and maternal education [69]. The notable disparities in health indicators observed between non-indigenous and indigenous children in Brazil underscore that these basic services are not yet as widely distributed in Brazil’s indigenous communities as they are in the rest of the country.

The poor sanitary conditions under which the majority of Brazil’s indigenous population lives, without access to human waste management, proper disposal of garbage, and safe drinking water at home, underlies the National Survey results indicating diarrhea as an important cause of recent hospitalization for indigenous children. This situation contrasts with the national Brazilian population, for which diarrhea is no longer considered a leading cause of child hospital admission in pediatric wards, even in the poorer regions of the country. For example, in the Brazilian Northeast, where in the 1990s diarrhea represented 57% of the total causes of hospitalization for children in the general population, this rate is now less than 15% [70]. These optimistic national results have been attributed to multiple sanitary interventions under way in Brazil for the last three decades, reduction in infant undernutrition, and widespread availability of oral rehydration therapy through the public healthcare system. According to Victora [71], this set of measures and interventions had such a positive impact on child health in Brazil that

“…*Anyone who has worked with child health in Brazil knows that these declines are real*. *Hospital admissions due to diarrhea have also dropped markedly in the poorest parts of the country*, *and it is now difficult*, *if not impossible*, *to teach our medical students the signs of acute dehydration in children*, *which once used to be a common finding in our outpatient and emergency services*”.

Unfortunately, the results of the National Survey do not permit the conclusion that the indigenous component of the Brazilian population has equal access to these important advances. The results of this National Survey and recent regional studies confirm that diarrhea remains a leading cause of hospitalization for indigenous children, being commonly associated with acute undernutrition and dehydration [25,26,72].

The National Survey represents a major step in documenting the health and nutrition conditions of indigenous peoples in Brazil. Its results provide for the first time the information necessary to characterize the health and nutrition profile of indigenous peoples in Brazil on a national scale. One of its most important potential contributions is to identify the social and health inequities that continue to exist among the indigenous peoples in Brazil, as compared to the non-indigenous population, despite the country having made major public health advances in recent decades. Such differences tend to increase the gap between indigenous and non-indigenous populations in Brazil in terms of health and nutrition indicators, as has been documented previously in numerous local case studies conducted in different regions of the country.

It is important to emphasize that despite its significance for characterizing the country’s national and regional indigenous populations, the design of the National Survey does not allow characterization of health conditions for specific indigenous ethnic groups. Although desirable, doing so would be a logistical challenge of enormous scale, considering that there are more than two hundred indigenous ethnicities in Brazil, some of which have populations of less than 200 people residing in remote and inaccessible locations.

## Conclusions and future directions

The overview of results presented in this article precedes a series of publications reporting on specific issues related to the demography and health of Brazil’s indigenous population based on the National Survey findings. Whereas the present article focuses on national and regional prevalence rates of major health and sanitation indicators, these future articles will report on more diverse sets of variables and will present statistical analyses to ascertain the associations between them. A first group of articles will address nutritional assessment of children, child anemia and associated factors, morbidity due to diarrhea and acute respiratory infection in children, and coverage and quality of prenatal care delivered to women. A second group of articles will address nutritional assessment of women, anemia and associated factors in women, blood pressure levels and hypertension in women, history of malaria in women and children, assessment of child BCG vaccination, and household dietary economies.

According to its original formulation, the National Survey was to focus on indigenous women and young children residing within federally recognized villages served by the Indigenous Healthcare Subsystem. As a result, certain segments of Brazil’s indigenous population were not included in the sample design, namely, men, adolescents, the elderly of both sexes, as well as Brazil’s self-identified indigenous population residing outside of the villages identified by FUNASA. Future nationally representative demographic and health surveys of indigenous peoples in Brazil should consider including these population segments, for which few data are currently available.

The primary motivation for publishing the present article, as well as the future articles mentioned above, is to augment the coverage of information deriving from national surveys in Brazil, which never before specifically addressed indigenous peoples. However, it is also hoped that the results presented here and to be published in the future will stimulate in-depth medium-scale surveys aiming to better characterize local health conditions in Brazil’s major indigenous cultural and geographical regions, as well as in its more populous ethnic groups. As argued by Jokisch and McSweeney [41] in an article reporting the results of a demographic and health survey of the Shuar people, a populous indigenous ethnic group in Ecuador, medium-scale regional surveys are an effective but under-utilized tool that have the potential to fill the gap between national surveys of indigenous populations, such as the one reported here, and local case studies, which are the type most frequently realized due to their lower costs and more modest logistical requirements. If the National Survey has the unique capacity to characterize indigenous health and sanitation conditions at a national and macro-regional scale in Brazil, medium-scale surveys will have the best potential to uncover the unique local circumstances that contribute to these patterns.

## Endnotes

^a^The objectives of the study and some aspects of its methodology, including the target population, the categories of variables collected, and stratification of the population into four geopolitical regions, were predefined through a public request for proposals by FUNASA, the government agency responsible for indigenous health services at the time of the study, and the World Bank. After the study, responsibility for indigenous health services and policies were assumed by the newly created Indigenous Health Secretariat (Secretaria de Saúde Indígena), Ministry of Health. ^b^FUNASA did not have an operational definition for the term “indigenous village,” apparently using it for any place groups of Indians lived, regardless of the existence of elements of social organization that could characterize a village or community. This created an important operational difficulty because the list included several enigmatic configurations of “villages,” including those without inhabitants (possibly already abandoned), those with only male inhabitants, those comprising small sectors of neighborhoods on the outskirts of larger cities, and those identified as transient camps within military installations, among others. These situations were verified to the extent possible before preparation of the final list of villages to be used for the sample selection. ^c^This cutoff point was selected because such small numbers of inhabitants would have overextended the available research budget despite not having a statistical bearing on the survey results nationally or by regional stratum. ^d^Due to droughts that routinely impact rural areas in the Northeast region, the Brazilian government promotes the construction of ponds to store rainwater or distributes water directly to communities in water trucks.

## Abbreviations

ABRASCO: Associação Brasileira de Saúde Coletiva; BMI: Body mass index; BP: Blood pressure; CI: Confidence interval; CONEP: Comissão Nacional de Ética em Pesquisa; ENSP: Escola Nacional de Saúde Pública; FUNAI: Fundação Nacional do Índio; FUNASA: Fundação Nacional de Saúde; IBGE: Instituto Brasileiro de Geografia e Estatística; JNC: The Joint National Committee on Detection, Evaluation, and Treatment of High Blood Pressure; MS: Ministério da Saúde; SPSS: Statistical Package for the Social Sciences; UN: United Nations.

## Competing interests

The authors declare that they have no competing interests.

## Authors’ contributions

All authors participated in formulating the research concept and design. CEAC, RVS, JRW, AMC, ER, LG, and BLH participated in field data collection. MCS, AMC, RVS, and BLH conducted statistical analyses. CEAC, RVS, JRW, and AMC wrote the manuscript and all other authors read and commented on the manuscript. The final paper submitted for publication was read and approved by all authors.

## Authors’ information

CEAC was coordinator-general of the First National Survey of Indigenous People’s Health and Nutrition. MLF represented the University of Gothenburg in the research consortium with ABRASCO. AMC, BLH, MLF, and RVS participated in coordinating the study. MCS coordinated data entry and database organization. JRW, LG, and ER were senior field researchers.

## Pre-publication history

The pre-publication history for this paper can be accessed here:

http://www.biomedcentral.com/1471-2458/13/52/prepub
